# Venetoclax or Ruxolitinib in Pre-Transplant Conditioning Lowers the Engraftment Barrier by Different Mechanisms in Allogeneic Stem Cell Transplant Recipients

**DOI:** 10.3389/fimmu.2021.749094

**Published:** 2021-09-24

**Authors:** Joanne E. Davis, Kelei Du, Mandy J. Ludford-Menting, Ashvind Prabahran, Eric Wong, Nicholas D. Huntington, Rachel M. Koldej, David S. Ritchie

**Affiliations:** ^1^ Australian Cancer Research Foundation (ACRF) Translational Research Laboratory, The Royal Melbourne Hospital, Melbourne, VIC, Australia; ^2^ The Department of Medicine, The University of Melbourne, Melbourne, VIC, Australia; ^3^ School of Medicine, Tsinghua University, Beijing, China; ^4^ Molecular Immunology Division, The Walter and Eliza Hall Institute of Medical Research, Melbourne, VIC, Australia; ^5^ The Department of Medical Biology, The University of Melbourne, Melbourne, VIC, Australia; ^6^ Clinical Haematology and Bone Marrow Transplantation Service, The Royal Melbourne Hospital, Melbourne, VIC, Australia; ^7^ Department of Biochemistry and Molecular Biology, Biomedicine Discovery Institute, Monash University, Clayton, VIC, Australia; ^8^ oNKo-Innate Pty Ltd., Moonee Ponds, VIC, Australia

**Keywords:** venetoclax, ruxolitinib, reduced intensity conditioning, graft versus tumour effect, MHC class-II, graft versus host disease, allogeneic stem cell transplantation

## Abstract

Allogeneic stem cell transplantation (alloSCT) is utilised to cure haematological malignancies through a combination of conditioning regimen intensity and immunological disease control *via* the graft versus tumour (GVT) effect. Currently, conventional myeloablative chemotherapeutic or chemoradiation conditioning regimens are associated with significant side effects including graft versus host disease (GVHD), infection, and organ toxicity. Conversely, more tolerable reduced intensity conditioning (RIC) regimens are associated with unacceptably higher rates of disease relapse, partly through an excess incidence of mixed chimerism. Improvement in post-alloSCT outcomes therefore depends on promotion of the GVT effect whilst simultaneously reducing conditioning-related toxicity. We have previously shown that this could be achieved through BCL-2 inhibition, and in this study, we explored the modulation of JAK1/2 as a strategy to lower the barrier to donor engraftment in the setting of RIC. We investigated the impact of short-term treatment of BCL2 (venetoclax) or JAK1/2 (ruxolitinib) inhibition on recipient natural killer and T cell immunity and the subsequent effect on donor engraftment. We identified striking differences in mechanism of action of these two drugs on immune cell subsets in the bone marrow of recipients, and in the regulation of MHC class-II and interferon-inducible gene expression, leading to different rates of GVHD. This study demonstrates that the repurposed use of ruxolitinib or venetoclax can be utilised as pre-transplant immune-modulators to promote the efficacy of alloSCT, whilst reducing its toxicity.

## Introduction

Allogeneic stem cell transplantation (alloSCT) is used to cure a range of haematological malignancies in part through the induction of the graft versus tumour (GVT) response mediated by engrafted donor immunity (1). Myeloablative conditioning (MAC) regimens have been the mainstay of allogeneic transplantation and produce reliable donor T cell engraftment but are associated with transplant-related toxicity and graft-versus host disease (GVHD), which collectively contribute to a transplant-related mortality of 20% in most series (2). In order to mitigate these toxicities in older patients or in those with comorbidities, a range of reduced-intensity conditioning (RIC) regimens have been employed over the last 20 years of alloSCT practice and now accounts for nearly two thirds of all transplant conditioning regimens (3, 4). However, RIC is often associated with mixed donor cell chimerism and a concurrent reduction in the GVT effect (5–7). Therefore, novel approaches to conditioning are required to enhance and maintain donor engraftment and GVT effect following RIC, whilst avoiding the toxicity and mortality rates associated with MAC.

We have previously shown that following RIC, residual recipient immunity acts as a barrier to donor engraftment that can be overcome by the addition of targeted therapy to RIC regimens (8, 9). Importantly, the brief pharmacological inhibition of BCL2 using venetoclax prior to RIC in mice resulted in depletion of residual recipient immunity and subsequent rapid donor cell engraftment in most recipients. Additionally, an absence of inflammatory cytokine production and avoidance of GVHD onset was observed, whilst the GVT effect against acute myeloid leukaemia (AML) was maintained (8). The Janus Kinase (JAK) 1/2 inhibitor ruxolitinib first showed its ability to profoundly decrease inflammatory cytokines in the treatment of myelofibrosis (10), and was the first drug to be approved by the FDA for the treatment of steroid-refractory GVHD (11, 12) *via* reduction of inflammatory cytokine production by T, NK and dendritic cells [reviewed by (13)]. Ruxolitinib has been established as an important and safe component of salvage therapy for the treatment of steroid-refractory acute GVHD (14, 15).

Based on our observations that donor engraftment and anti-tumour efficacy of alloSCT following RIC can be enhanced through venetoclax-induced depletion of residual recipient immunity, we hypothesised that suppression of inflammatory cytokines using ruxolitinib may also lower the engraftment barrier in RIC and result in similar post-alloSCT outcomes. In this paper we explored the effects of ruxolitinib in a RIC alloSCT model and compared the mechanisms to those observed in a venetoclax-containing RIC regimen.

## Materials and Methods

### Experimental Mice

Experimental mice were specific-pathogen-free (SPF) and all animal work was conducted with standard operating procedures approved by institutional animal ethics committees. The alloSCT experiments were performed either at the Biological Research Facility of the Victorian Comprehensive Cancer Centre (VCCC) or the Bioservice Department of the Walter and Eliza Hall Institute of Medical Research (WEHI). IL-15 KO (16) mice with C57BL/6 background were bred and used at WEHI. All mice used as recipients for transplantation were 6-14 weeks of age when the experiments were set up. BALB/c donors were purchased at 6-8 weeks of age, and sex-matched to the recipients.

The MHC-mismatched allogeneic SCT (alloSCT) model used mice with C57BL/6 background (H-2Kb) as recipients and BALB/c (H-2Kd) allogeneic donors. Recipients (n=6/group) received split-dose total body irradiation (TBI) by a cobalt-60 irradiator, of either myeloablative (MAC) (2 × 550 rad) or reduced intensity conditioning (RIC) dose (2 × 400 rad) delivered two hours apart. 7.5 x10^6^ bone marrow (BM) cells and 1 x10^6^ T cells (splenic CD4+ T cells and CD8+ T cells mixed in a 2:1 ratio) from BALB/c donors were intravenously injected into recipients at least two hours after irradiation. AlloSCT recipients were monitored regularly for body weight and clinical scores based on posture, activity, and eye appearance [scores of 3 for each, adapted from (17)], and were humanely killed once 20% of initial body weight loss or clinical scores of 4 were reached. Donor haematopoietic cell engraftment examined the donor:recipient (H-2Kd/H-2Kb) ratio within peripheral blood. Donor cell engraftment and cell profiles within organs were also analysed at the experimental endpoint.

### Chemical Compounds

The BCL2 inhibitor venetoclax and JAK1/2 inhibitor ruxolitinib (SelleckChem, Houston, TX) were used to treat C57BL/6 WT mice for two days prior to alloSCT. Venetoclax (100 mg/kg) and its vehicle (60% phosal R 50 PG (Merck, Germany), 30% polyethylene glycol (PEG) 400 (Merck, Germany), 10% ethanol) were administered by oral gavage once daily for two days, with a cumulative total dose of 4 mg. Ruxolitinib (180 mg/kg) and its vehicle (2% DMSO, 30% PEG 300 (Merck, Germany), ddH2O) were administered twice a day by oral gavage for two days, with a cumulative total dose of 14.4 mg.

### GVHD Histology

Recipient rectum and colon tissue without stool were preserved in 10% neutral buffered formalin (Merck, Germany), and Haemotoxylin and Eosin (H&E) staining and digital images *via* a 20x slide scanning were processed by the Histology Department of WEHI. Histology scores were given to the gut tissues according to the number of apoptotic cells, mucosal integrity, and lymphocyte infiltration (each scored out of 3), by an independent, blinded pathologist.

### Graft Versus Tumour Model

Wild type C57BL/6 mice were inoculated with 0.8 × 10^6^ (mixed lineage leukaemia) GFP+ MLL-AF9 acute myeloid leukaemia (AML) cells. After 8 days, mice were treated with ruxolitinib (180 mg/kg) by oral gavage twice daily for two days. The following day mice were irradiated with RIC and injected with 7.5 x10^6^ BM cells and 1 x10^6^ T cells from BALB/c donors. Mice were monitored regularly for body weight, clinical scores, donor cell engraftment and AML burden in the blood, and were killed after 21 days post alloSCT.

### Flow Cytometric Analysis

Peripheral blood samples were collected with or without EDTA to separate blood cells and plasma/serum, which were stored at -20°C for cytokine analysis. Single cell suspension of splenocytes, peripheral blood, BM and liver cells (purified using a 33.75% Percoll R Density Gradient (GE Healthcare, Sweden), were resuspended in FACS buffer (PBS + 2% FCS) after red blood cell lysis. Cells were mixed with FACS buffer containing 1/100 mouse Fc blocking antibody (purified Rat anti-Mouse CD16/CD32, 2.4G2, BD Biosciences, San Jose, CA) and specific antibody cocktail on ice for 30 minutes. After unbound antibodies were washed away, cells were fixed in 2% paraformaldehyde and analysed on a BD LSRFortessaII (BD Biosciences). The following antibodies were used to identify donor cells (H-2Kd; e450, SF1-1.1.1), recipient cells (H-2Kb; PE, AF6-88.5), leukocytes (CD45; BV611, 30-F11), T cells (CD3; BV785, 17A2), CD4+ T cells (CD4; BUV805/APCe780, GK1.5), CD8+ T cells (CD8a; PerCP-Cy5.5/PE-Cy7/BUV395, 53-6.7), memory T cells (CD44 and CD62L; APC-Cy7, IM7; PE-Cy7, MEL-14), B cells (CD19; BV711, ID3), myeloid cells (CD11b and Ly6C/G; BUV395, M1/70; APC, RB6-8C5), NK cells (NK1.1, NKp46 and CD49b; BV650, PK136; PECy7, 29A1.4; BB700, HMα2), mature NK cells (CD11b and CD27; BV605, M1/70; APC-e780; LG.7F9), ILC1s (NK1.1, NKp46 and CD49a; BV650, PK136; PECy7, 29A1.4; BV711, Ha31/8). All mAbs were from BD Biosciences, except for CD27, H2Kd and NKp46 (Thermo Fisher, Waltham, MA).

FlowJo (BD Biosciences, San Jose, CA) analysis was used to identify NK (NK1.1+CD3-), cNK (NKp46+CD49b+), ILC1s (NKp46+CD49a+), CD4 (CD3+CD4+) and CD8 (CD3+CD8+) T cells, B cells (CD19+), and granulocytes (CD11b+Ly6G+). Phenotypic subsets were characterised by the expression of the following cell surface markers: M1 Mature (CD11b+CD27+), M2 mature (CD11b+CD27-) and immature (CD11b-CD27+) NK cells, naive (N; CD44-CD62L+); central memory (CM; CD44+CD62L+); effector memory (EM; CD44+CD62L-) CD4 and CD8 T cells; and virtual memory (VM; CD8+CD44+CD62L+CD49d+) T cells.

### Cytometric Bead Array

Plasma/serum samples from specific timepoints post-alloSCT were tested using the Cytometric Bead Array (CBA) Mouse Inflammation Kit (BD Biosciences, San Jose, CA) as per manufacturer’s instructions. The CBA was analysed using FCAP Array v3.0 Analysis Software (BD Biosciences, San Jose, CA).

### Gene Expression Analysis

Total BM RNA was extracted from cohorts of mice (n=3-4) days 1, 3 and 7 post-drug treatment or from untreated controls using the Qiagen RNeasy Kit (Qiagen, Venlo, The Netherlands). Gene expression was determined using the NanoString Mouse PanCancer Immune Profiling Panel (NanoString Technologies, Seattle, WA) as per manufacturer’s instructions. All raw data was reviewed, and all samples in downstream analysis had no quality control flags and detection of at least 20% of probes. All experiments were normalised and analysed using nCounter Advanced Analysis (version 2.0.115; NanoString Technologies).

### Statistical Analysis

Statistical analysis was conducted using, unpaired T test, Mann-Whitney unpaired T test, Ordinary One-Way Anova Holm-Sidak’s multiple comparisons test, and Pearson’s Correlation coefficient as indicated, using GraphPad Prism V9.2.0 (San Diego, CA). Significance is indicated as follows: p<0.05(*), p<0.01(**), p<0.001(***), p<0.0001(****).

## Results

### Recipient NK and CD8+ T Cells Regulate Donor Cell Engraftment and Onset of Acute GVHD

**Figure 1:**
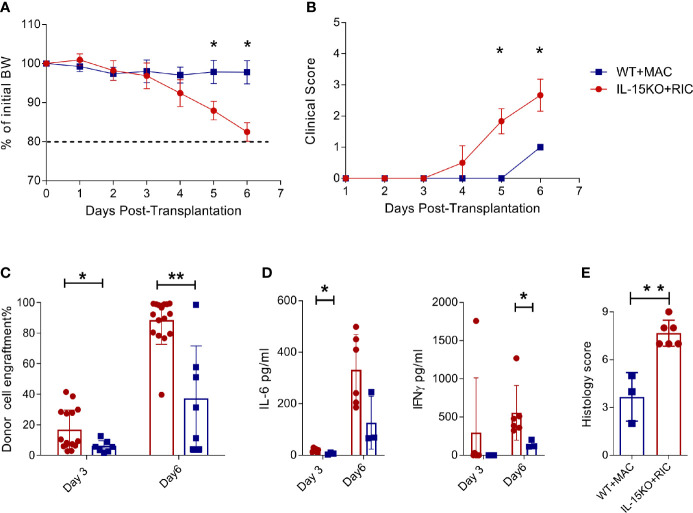
IL-15 KO + RIC alloSCT recipients develop hyperacute GVHD. C57BL/6 WT mice were irradiated with MAC, and IL-15 KO mice were irradiated with RIC, followed by alloSCT. Mice were monitored daily for **(A)** body weight and **(B)** clinical scores, and **(C)** donor cell engraftment and **(D)** plasma cytokine concentrations of IL-6 and IFNγ on days 3 and 6. **(E)** Mice were killed on day 6 post-alloSCT, and GVHD histology was conducted on gut tissue. Statistical analysis was performed using Mann-Whitney unpaired T test. *p < 0.05, **p < 0.01.

NK cells present an engraftment barrier in RIC treated mice (9), and NK cell survival is dependent on IL-15 signalling (18, 19). To explore the outcome of alloSCT in recipients in which the engraftment barrier was absent, we compared C57BL/6 WT mice transplanted using MAC compared with IL-15 KO mice transplanted using RIC. IL-15 KO mice lack mature NK cells and also have 10-fold fewer immature NK cells compared to WT mice (**Supplementary Figures 1A, B**). Within 3 days post alloSCT, IL-15 KO recipients developed rapid weight loss (**Figure 1A**) and high clinical GVHD scores (**Figure 1B**), and had to be killed by day 6 due to hyperacute GVHD. Donor cell engraftment was greater than 80% by day 6 post-transplant, and was accompanied by elevated IFNγ and IL-6 levels, and high GVHD histology scores in the gut (**Figures 1C–E**). Therefore, while IL-15 KO successfully removed the recipient-derived engraftment barrier, it was at the cost of unmitigated donor T cell expansion, cytokine production and onset of severe, fatal GVHD.

### Ruxolitinib Treatment Combined With RIC Reduces T and NK Cells and Allows Full Engraftment

**Figure 2:**
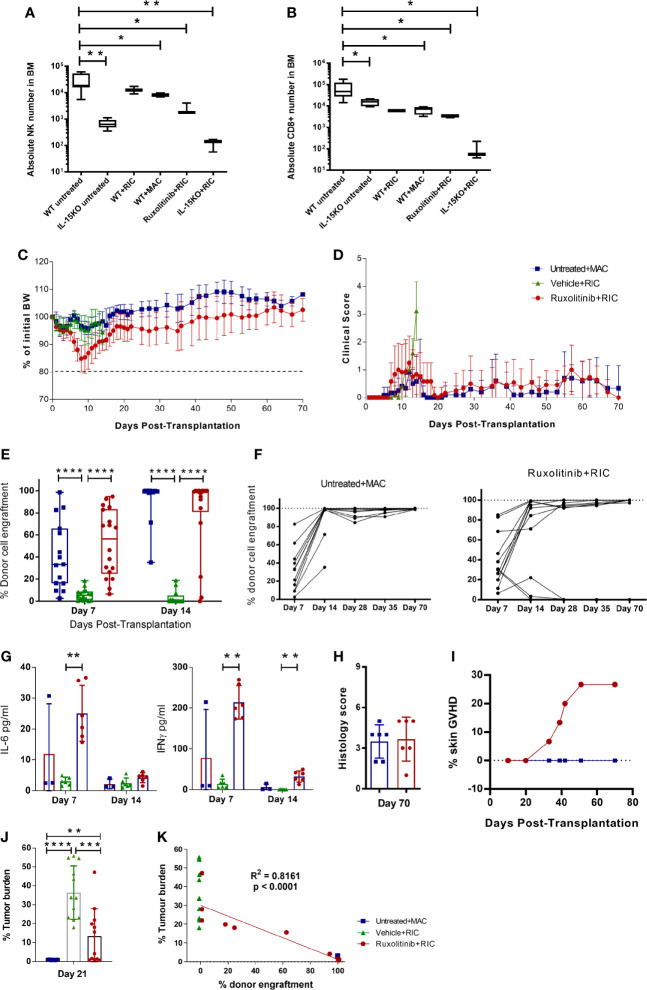
Ruxolitinib treatment in combination with RIC mediates rapid and long-term donor cell engraftment, and permits GVT responses. Untreated WT and IL-15KO mice were compared to WT mice treated with RIC or MAC; or WT mice treated with ruxolitinib prior to RIC, or IL-15 KO mice treated with RIC. Mice were killed four days after receiving irradiation, and the absolute number of **(A)** NK cells (NKp46+CD49b+) and **(B)** CD8 (CD3+CD8+) T cells in BM were compared between different cohorts of mice (n=3-9/group). C57BL/6 WT mice were treated with ruxolitinib or vehicle for two days, and the following day treated with RIC and alloSCT. Another cohort of untreated WT mice was treated with MAC and alloSCT. Mice were monitored for **(C)** body weight and **(D)** clinical scores up to 70 days post alloSCT. **(E, F)** Donor cell engraftment (H2Kd+ cells) was monitored on days 7, 14, 28, 35 and 70 post-alloSCT in blood samples (n= 18, data combined from 3 independent experiments). **(G)** Plasma cytokine concentration of IFNγ and IL-6 was measured in blood samples collected on days 7 and 14 post-alloSCT. **(H)** Mice were killed 70 days post-alloSCT, and GVHD histology was conducted on gut tissue. **(I)** Incidence of development of skin GVHD in ruxolitinib+RIC mice compared to untreated+MAC alloSCT recipients (n=15). Mice were injected i.v. with MLL-AF9 tumour cells, and 8 days later were treated with ruxolitinib or vehicle for two days, and the following day treated with RIC and alloSCT. Another cohort of untreated WT mice was treated with MAC and alloSCT (n=12/treatment group, data combined from 2 independent experiments). **(J)** Mice were killed 21 days after alloSCT, and tumour burden was measured as a percentage of MLL-AF9+ cells in the BM. **(K)** Tumour burden was compared to donor cell engraftment between the untreated+MAC, vehicle+RIC and ruxolitinib+RIC cohorts 21 days after alloSCT. R^2^ value indicates the correlation between tumour burden and donor cell engraftment in ruxolitinib+RIC alloSCT recipients. Statistical analysis was performed using Ordinary One-way Anova Holm-Sidak’s multiple comparisons test **(A, B)**, Mann-Whitney unpaired T test **(E–J)**, and Pearson’s Correlation coefficient **(K)**. *p < 0.05, **p < 0.01, ***p < 0.001, **** p < 0.0001.

To compare the levels of immune depletion during conditioning, we first investigated NK and T cell depletion post conditioning in WT and IL-15 KO mice. Mice were left untreated or given a MAC or RIC irradiation dose, and killed 4 days later to examine the absolute NK or CD8+ T cell numbers remaining in the BM. WT mice irradiated with RIC or MAC had a significant decrease in NK cells compared to untreated mice, but were still 10-fold higher than in untreated IL-15 KO mice (**Figure 2A**). In contrast, RIC or MAC treated WT mice had CD8+ T cell numbers similar to IL-15 KO untreated mice, and RIC treatment of IL-15 KO mice almost ablated both NK and CD8+ T cells in the BM (**Figure 2B**). In order to pharmacologically replicate the IL-15 KO phenotype, WT mice were treated with the JAK1/2 inhibitor ruxolitinib and RIC, resulting in a reduction of NK and CD8+ T cell numbers comparable to those in untreated IL-15 KO mice.

Next, we determined if transient inhibition of JAK1/2 was able to replicate the lowered engraftment barrier seen in the IL-15 KO mice, while maintaining the GVHD control of the WT mice. Depletion of recipient immunity using combination ruxolitinib and RIC was well tolerated, with minimal weight loss and low clinical scores recovering within 2 weeks post-alloSCT (**Figures 2C, D**). Mice treated with ruxolitinib and RIC engrafted by 7 days post-alloSCT, unlike vehicle and RIC treated mice which rejected the graft (**Figure 2E**). After 14 days post-alloSCT, over 80% of ruxolitinib treated mice had engrafted with donor cells, which was comparable to the MAC treated cohort, and was maintained at 70 days (**Figures 2E, F**). In comparison, the mice treated with vehicle and RIC uniformly rejected the graft and were killed on day 14 upon developing increasing clinical scores due to anaemia (**Figures 2D, E**). Donor cell engraftment in ruxolitinib treated mice was associated with moderate IFNγ and low IL-6 levels in the plasma at day 7 post-alloSCT, which abated by day 14 (**Figure 2G**). Mice killed at 70 days post-alloSCT did not develop the early gut GVHD (**Figure 2H**) that was seen in the IL-15 KO mice, however between day 30-50 post-alloSCT skin GVHD developed in approximately 25% of mice treated with ruxolitinib and RIC, which was not observed in WT mice treated with MAC (**Figure 2I**). Mice with skin GVHD had to be killed due to ulceration of the skin which developed after localised fur loss on the hind flanks.

We previously established a pre-clinical model in C57BL/6 WT mice of AML (MLL-AF9) matched to the BALB/c allogeneic donor haplotype (H2kd+), to examine the effect of venetoclax treatment with RIC on donor cell engraftment and subsequent GVT effect (8). In this study, ruxolitinib treatment improved GVT responses compared to vehicle treated controls, with some mice showing tumour control comparable to MAC treated mice (**Figure 2J**). The level of tumour response strongly correlated with the level of donor cell engraftment, with mice that had full engraftment showing complete tumour control, whereas mice that rejected the graft or had mixed chimerism (5-90% donor cells) had impaired GVT responses (**Figures 2J, K**). Overall, this suggests that transient inhibition of JAK1/2 signalling reduces the engraftment barrier presented by residual recipient NK and T cells, allowing full donor engraftment, whilst improving GVT and decreasing priming of acute GVHD onset.

### Donor Cell Engraftment Is Dependent on Recipient Pre-SCT Conditioning

**Figure 3:**
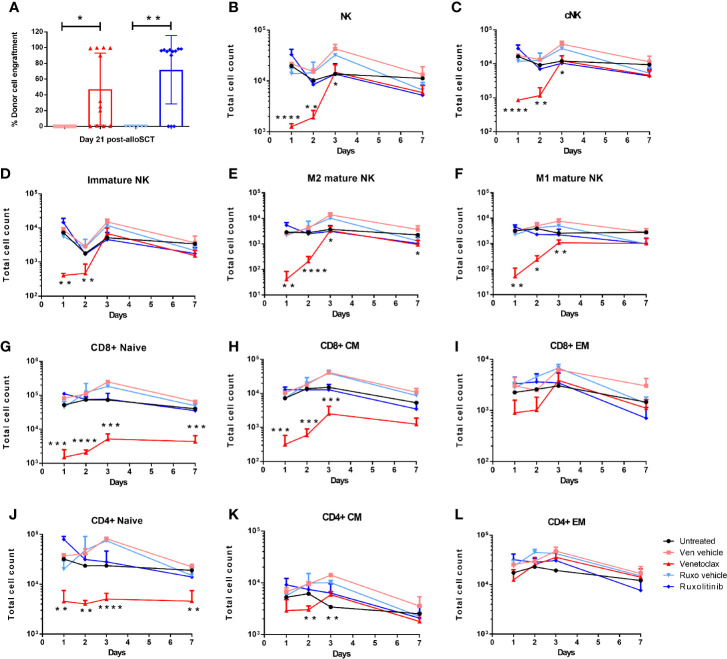
Donor cell engraftment is dependent on recipient pre-alloSCT irradiation dose and treatment with BCL2 or JAK1/2 inhibitors. WT mice were treated with venetoclax or ruxolitinib, or their respective vehicle for two days. The following day mice were treated with RIC and alloSCT. **(A)** Donor cell engraftment (H2kd+ cells) was measured in the blood at day 21 post-alloSCT. WT mice were treated with venetoclax or ruxolitinib, or their respective vehicle for two days. Mice (n=3-4/group) were killed on days 1, 2, 3, and 7, and BM was harvested and analysed by flow cytometry for the absolute number of **(B–F)** NK cells (NK1.1+CD3-), cNK (NKp46+CD49b+), M1 mature (CD11b+CD27+), M2 mature (CD11b+CD27-) and immature (CD11b-CD27+) NK cells; **(G–L)** naive (N; CD44-CD62L+); central memory (CM; CD44+CD62L+); effector memory (EM; CD44+CD62L-) CD4+ and CD8+ T cells. Data is representative of 3 independent experiments. Statistical analysis was performed using unpaired T test. *p < 0.05, **p < 0.01, ***p < 0.001, ****p < 0.0001.

Our previous work has shown that pre-treatment of alloSCT recipients with short-term pharmacological inhibition of BCL2 (venetoclax) in combination with RIC permits rapid donor cell engraftment in a high percentage of mice, without graft rejection or GVHD (8). Approximately 80% of WT mice administered venetoclax for two days immediately prior to RIC and alloSCT obtained donor cell engraftment within 14 days, however approximately 40% of venetoclax-treated mice developed graft rejection after an initial period of donor engraftment (**Figure 3A** (8). In contrast, over 80% of ruxolitinib-treated alloSCT recipients retained long-term donor engraftment (**Figures 2F** and **3A**).

To understand the mechanism of recipient immune cell inhibition with venetoclax and ruxolitinib, WT mice were treated for two days with either venetoclax, ruxolitinib, or their respective vehicles, and then killed on day 1, 2, 3, and 7 to profile immune cell subsets in BM, spleen and liver. In contrast to ruxolitinib, venetoclax treatment rapidly depleted NK cells, including conventional (NKp46+CD49b+), immature (CD11b-CD27+), and M1 (CD11b+CD27+) and M2 (CD11b+CD27-) mature NK cells from the spleen and liver, and most strikingly from the BM (**Figures 3B–F**, **Supplementary Figure 2A**). Furthermore, venetoclax rapidly depleted CD8+ and CD4+ naïve (CD62L+CD44-) and CD8+ central memory (CD62L+CD44+) T cells in the BM, spleen and liver (**Figures 3G–L**, **Supplementary Figure 2B**). Therefore, BCL2 inhibition affected recipient immune cell function by rapidly depleting, most notably in the BM, CD8+ naïve and central memory T cells, CD4+ naïve T cells and NK cells, whereas JAK1/2 inhibition had no significant impact on immune cell subsets.

### Venetoclax and Ruxolitinib Differentially Affect MHC-II and IFN Gene Expression

**Figure 4:**
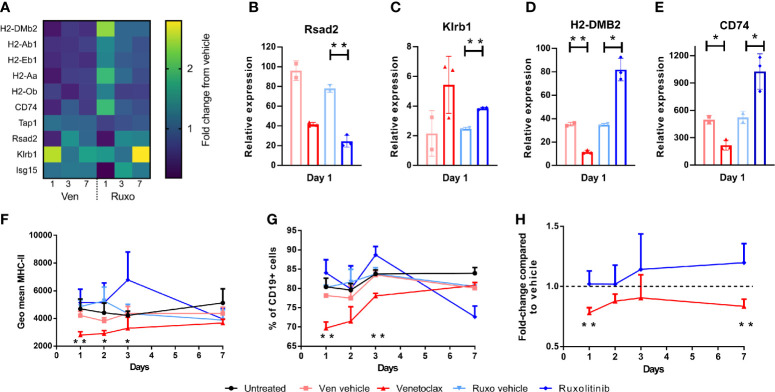
Venetoclax and Ruxolitinib differentially affect MHC class-II and IFN-inducible gene expression. WT mice were treated with venetoclax or ruxolitinib, or their respective vehicle for two days. Mice (n=3-4/group) were killed on days 1, 3, and 7, and gene expression was determined from BM RNA using the NanoString Mouse PanCancer Immune Profiling Panel. **(A)** Heat map of relative gene expression of *H2-DMb2, H2-Ab1, H2-Eb1, H2-Aa, H2-Ob, CD74, Tap1, Rsad2, Klrb1* and *Isg15* from venetoclax and ruxolitinib-treated mice. Relative expression of *Rsad2, Klrb1, H2-DMB2* and *CD74* **(B–E)** was compared between venetoclax or ruxolitinib-treated mice and their respective vehicle on day 1 post-treatment. The geometric mean of MHC-II expression on CD19+ B cells **(F)**, percentage of CD19+ B cells expressing MHC-II **(G)**, and the fold-change of MHC-II expression on total CD45+ BM cells from venetoclax or ruxolitinib-treated mice was compared to each vehicle control group **(H)**. Statistical analysis was performed using Mann-Whitney unpaired T test **(B–E)**, and unpaired T test **(F–H)**. *p < 0.05, **p < 0.01.

The absence of change in cell subsets in ruxolitinib treated mice, despite the improved engraftment seen when these mice are used as alloSCT recipients, suggested that ruxolitinib may supress immune cell function rather than directly deplete immune cells as seen with venetoclax. Therefore, gene expression analysis was performed on BM samples from venetoclax, ruxolitinib, vehicle treated, and untreated C57BL/6 WT mice collected at days 1, 3 and 7 post-treatments, to examine which immune pathways were impacted by drug treatment. Several MHC-II genes were differentially affected by venetoclax or ruxolitinib treatment, including *H2-DMb2*, *H2-Ab1*, *H2-Eb1*, *H2-Aa*, *H2-Ob* and *CD74* (**Figure 4A**). Venetoclax downregulated relative gene expression of MHC-II genes, in contrast to ruxolitinib treatment which resulted in MCH-II upregulation. The expression of the interferon (IFN) genes *Rsad2*, *Ifit3*, *Ifnb1*, *Ifna1*, *Oas2*, *Isg15*, *Klrb1* and *Ifng* were also altered after drug treatment (**Figure 4A**). Both venetoclax and ruxolitinib treatment downregulated *Rsad2* expression which encodes Radical S-adenosyl methionine domain containing 2 (Rsad2) protein, an IFN-inducible virus inhibitory protein involved in CD4+ T cell activation (20) (**Figure 4B**). *Klrb1* encoding killer cell lectin-like receptor subfamily B member 1 (KLRB1), which inhibits IFNg production by NK cells (21), was upregulated by both venetoclax and ruxolitinib treatment (**Figure 4C**). As described above, *H2-DMB2* and *CD74* expression were downregulated by venetoclax, and upregulated by ruxolitinib (**Figures 4D, E**). The MHC-II-associated genes regulate antigen expression, and therefore likely alter alloantigen presentation in the intestinal epithelium after alloSCT (22). Subsequent flow cytometry analysis confirmed that cell surface MHC-II expression on BM CD19+ B cells, and the percentage of B cells expressing MHC-II was decreased for several days in venetoclax-treated mice, compared to untreated or vehicle treated mice, whereas expression of MHC-II increased in ruxolitinib-treated mice (**Figures 4F, G**). Furthermore, the changes to MHC-II expression were replicated in total BM CD45+ cells, as compared to each vehicle control (**Figure 4H**). Collectively, the differential effects of venetoclax and ruxolitinib on both cell type and gene expression demonstrate that these drugs work *via* different mechanisms and therefore lead to different impacts on transplant outcome when combined with RIC.

## Discussion

Successful donor engraftment in an alloSCT recipient requires that the conditioning regimen adequately suppresses recipient immunity to prevent donor cell rejection. This is reliably achieved in most recipients with intensive MAC regimens but at the cost of mortality and morbidity (2). Conversely, RIC regimens are safer, but associated with a higher incidence of mixed chimerism, increased rates of graft loss and poorer induction of the GVT effect with a resultant excess of relapse and poorer overall survival (5–7). Augmentation of existing RIC regimens has not been associated with improved outcomes, and strategies directed at improved donor T cell engraftment and promotion the GVT effect have been advocated (23).

We hypothesised that donor engraftment and subsequent GVT rates achieved by RIC could be improved by additional suppression of recipient immunity through either lymphocyte depletion or cytokine inhibition with either BCL2 or JAK inhibition. Similarly, we reasoned that by avoiding the tissue damage and inflammatory cytokine production associated with MAC and further suppressing JAK/STAT dependant cytokine production, in particular IL6, the rates of GVHD onset may be reduced in alloSCT recipients (24, 25).

In this study we first examined how the absence of recipient T and NK cells due to IL-15 deficiency would impact on donor cell engraftment following RIC. The resulting hyperacute, lethal gut GVHD observed in IL-15 KO recipients indicated that residual recipient immunity is necessary to prevent uncontrolled donor homeostatic T cell proliferation, activation and inflammatory cytokine production. Given the role for residual post-conditioning recipient immunity in controlling donor engraftment, we hypothesised that a brief period of venetoclax or ruxolitinib treatment added to RIC would provide a sufficient period of immunosuppression to promote donor engraftment, whilst not full removing the regulator function of residual recipient immunity. We identified that venetoclax rapidly depleted naïve and central memory CD4+ and CD8+ T cells, NK cells, and VM T cells in the BM, spleen and liver, and we have previously demonstrated that the combination of venetoclax and RIC results in donor engraftment and GVT without the onset of GVHD (8). The incorporation of ruxolitinib into RIC of WT alloSCT recipients also resulted in NK and CD8+ T cell depletion in BM similar to that induced by MAC, and resulted in similar donor engraftment rates and associated GVT responses as seem with the MAC and ventoclax + RIC combination. However, unlike venetoclax + RIC, the ruxolitinib-containing RIC regimen did not fully avoid the onset of chronic GVHD as skin chronic GVHD was observed 1-2 months after alloSCT.

Further exploration of the venetoclax or ruxolitinib treatment of alloSCT recipients identified significant differences in gene expression within the BM of recipient mice. Reduced MHC-II expression was observed in the BM following venetoclax treatment prior to alloSCT. In contrast, MHC-II expression increased in the BM of ruxolitinib treated mice, whilst IFN gene expression decreased transiently. Ruxolitinib therapy for two days prior to transplant was insufficient to suppress IFNγ expression in the first 7 days post alloSCT (**Figure 2G**). The variation between the gene expression changes seen between venetoclax and ruxolitinib therapy is important as IFNγ-dependent MHC-II expression in recipient tissues and subsequent activation of donor CD4+ T cells is now recognised as a key priming event in the onset of GVHD (22) and may explain, in part, why ruxolitinib + RIC treated recipients developed late skin GVHD.

In our model, despite the early IFNγ cytokine rebound observed after ruxolitinib-containing RIC and the high levels of donor cell engraftment achieved by this regimen, acute GVHD was not observed. This likely reflects the absence of GVHD-promoting gut inflammation that is associated with MAC. These observations suggest that by avoiding gut toxicity through the use of ruxolitinib + RIC, acute GVHD will not be primed even following high levels of donor T cell engraftment. Although other contributors to the prevention of GVHD onset including ruxolitinib-induced decrease in dendritic cell activation (26) cannot be excluded. The potential for ruxolitinib therapy to reduce inflammatory cytokine production has resulted in pilot studies exploring its ability to improve engraftment, avoid GVHD, and replace conventional GVHD prophylaxis (27). To date, studies of this approach have been small and although associated with likely lower rates of GVHD, ongoing ruxolitinib therapy may be limited by viral activation and post-transplant cytopenias (28, 29). In contrast, our approach of transiently lowering the engraftment barrier by a short exposure of ruxolitinib prior to donor cell infusion may provide an opportunity to optimise donor engraftment, maintain GVL and avoid GVHD onset, whilst avoiding the toxicity of continuous ruxolitinib exposure.

Overall, whilst either of the targeted therapies venetoclax or ruxolitinib are able to promote increased donor engraftment in the setting of RIC and thereby avoid the toxicity and GVHD-priming effects of MAC, the mechanism of action of venetoclax including its ability to reduce MHC-II expression, added to RIC seems best placed as the combination to take forward for clinical application in order to realise the GVT benefits of alloSCT, whilst avoiding GVHD.

## Data Availability Statement

The datasets presented in this study can be found in online repositories. The names of the repository/repositories and accession number(s) can be found below: https://www.ncbi.nlm.nih.gov/, GSE181060.

## Ethics Statement

The animal study was reviewed and approved by Walter and Eliza Hall Institute of Medical Research Animal Ethics Committee (AEC 12.08), and Peter MacCallum Cancer Centre Animal Ethics Committee (E607).

## Author Contributions

JD, KD, ML-M, AP, and RK performed experiments and analysis of results. NH provided the IL-15 KO mice. AP, EW, and NH contributed to project discussion. RK, JD, NH, and DR designed the study. All authors contributed to the article and approved the submitted version.

## Funding

This study was supported by a Cancer Council Victoria grant-in-aid (APP1145730) and Royal Melbourne Hospital grant-in-aid (GIA-109-2019).

## Conflict of Interest

DR received honoraria and research funding from Novartis. NH is a founder and shareholder of oNKo-Innate Pty Ltd.

The remaining authors declare that the research was conducted in the absence of any commercial or financial relationships that could be construed as a potential conflict of interest.

## Publisher’s Note

All claims expressed in this article are solely those of the authors and do not necessarily represent those of their affiliated organizations, or those of the publisher, the editors and the reviewers. Any product that may be evaluated in this article, or claim that may be made by its manufacturer, is not guaranteed or endorsed by the publisher.

## Supplementary Material

Supplementary Figure 1 IL-15 knockout mice have reduced NK and CD8+ T cells in the bone marrow. Untreated IL-15KO mice and WT C57BL/6 mice aged 8-12 weeks (n=6) were killed and cell profiles of BM were tested by flow cytometry. **(A)** The absolute number of NK cells (NKp46+CD49b+), ILC1s (NKp46+CD49a+), CD4 (CD3+CD4+) and CD8 (CD3+CD8+) T cells, B cells (CD19+), and granulocytes (CD11b+Ly6G+) were compared between WT and IL-15 KO mice. **(B)** The absolute number of mature (CD11b+CD27+/-) and immature (CD11b-CD27+) NK cells, naive (N; CD44-CD62L+); central memory (CM; CD44+CD62L+); effector memory (EM; CD44+CD62L-) CD4 and CD8 T cells; and virtual memory (VM; CD8+CD44+CD62L+CD49d+) T cells were compared between WT and IL-15 KO mice. Statistical analysis was performed using Mann-Whitney unpaired T test.

Supplementary Figure 2 Venetoclax treatment decreases NK and CD8+ T cells in the BM, spleen and liver. WT mice were treated with venetoclax or ruxolitinib, or their respective vehicle for two days, were killed the following day and the BM, spleen and liver was harvested and analysed by flow cytometry for the absolute number of **(A)** NK and **(B)** CD8+ T cells. Data is representative of 3 independent experiments. Statistical analysis was performed using Mann-Whitney unpaired T test.
