# Supplementary figures and images for: Venetoclax or Ruxolitinib in Pre-Transplant Conditioning Lowers the Engraftment Barrier by Different Mechanisms in Allogeneic Stem Cell Transplant Recipients
Source: Front Immunol. 2021 Sep 24;12:749094. doi: 10.3389/fimmu.2021.749094 (PMC8498041; doi:10.3389/fimmu.2021.749094)

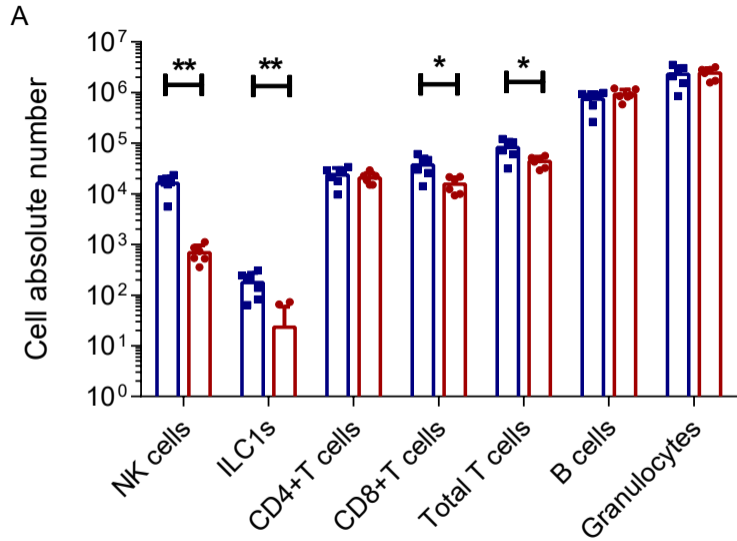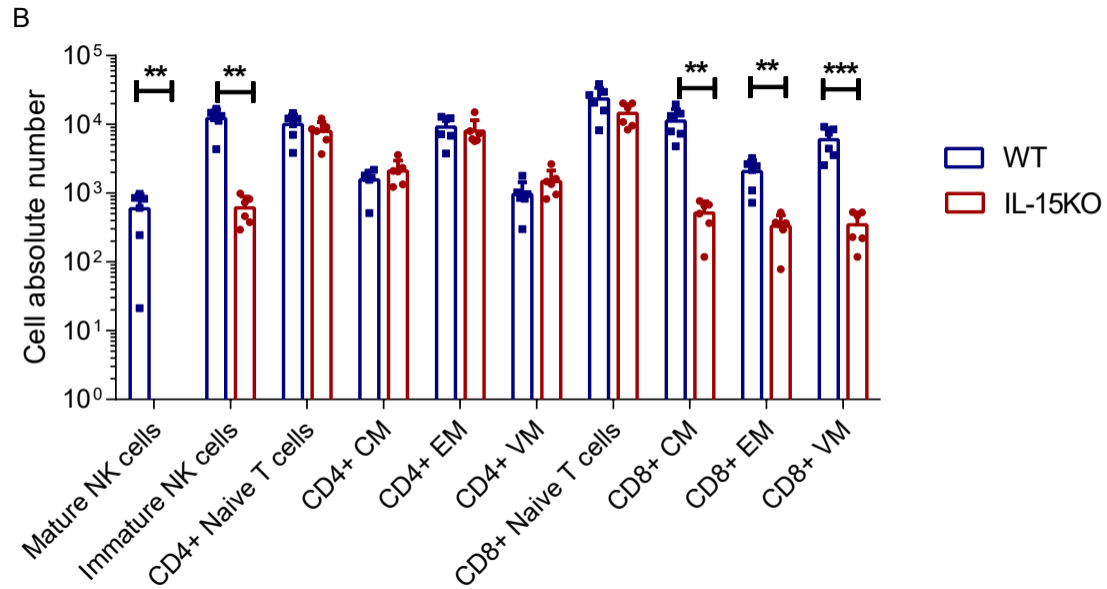

Supplement: Supplementary Figure 1 — IL-15 knockout mice have reduced NK and CD8+ T cells in the bone marrow. Untreated IL-15KO mice and WT C57BL/6 mice aged 8-12 weeks (n=6) were killed and cell profiles of BM were tested by flow cytometry. (A) The absolute number of NK cells (NKp46+CD49b+), ILC1s (NKp46+CD49a+), CD4 (CD3+CD4+) and CD8 (CD3+CD8+) T cells, B cells (CD19+), and granulocytes (CD11b+Ly6G+) were compared between WT and IL-15 KO mice. (B) The absolute number of mature (CD11b+CD27+/-) and immature (CD11b-CD27+) NK cells, naive (N; CD44-CD62L+); central memory (CM; CD44+CD62L+); effector memory (EM; CD44+CD62L-) CD4 and CD8 T cells; and virtual memory (VM; CD8+CD44+CD62L+CD49d+) T cells were compared between WT and IL-15 KO mice. Statistical analysis was performed using Mann-Whitney unpaired T test. [file DataSheet_1.pdf]

A

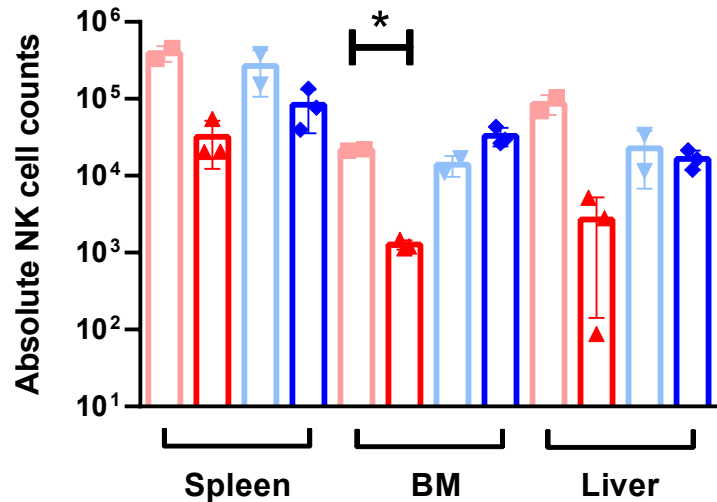

B

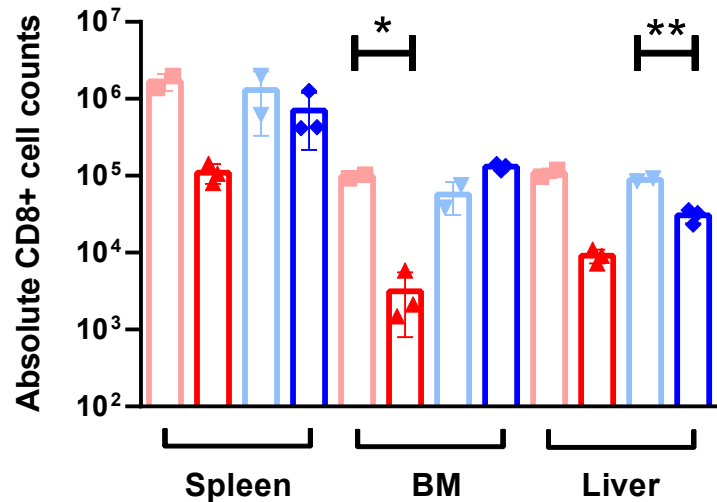

Supplement: Supplementary Figure 2 — Venetoclax treatment decreases NK and CD8+ T cells in the BM, spleen and liver. WT mice were treated with venetoclax or ruxolitinib, or their respective vehicle for two days, were killed the following day and the BM, spleen and liver was harvested and analysed by flow cytometry for the absolute number of (A) NK and (B) CD8+ T cells. Data is representative of 3 independent experiments. Statistical analysis was performed using Mann-Whitney unpaired T test. [file DataSheet_2.pdf]
